# Spleen Stiffness Is Superior to Liver Stiffness for Predicting Esophageal Varices in Chronic Liver Disease: A Meta-Analysis

**DOI:** 10.1371/journal.pone.0165786

**Published:** 2016-11-09

**Authors:** Xiaowen Ma, Le Wang, Hao Wu, Yuemin Feng, Xibiao Han, Haoran Bu, Qiang Zhu

**Affiliations:** 1 Department of Gastroenterology, Shandong Provincial Hospital Affiliated to Shandong University, Jinan, Shandong, China; 2 Shandong Provincial Engineering and Technological Research Center for Liver Diseases Prevention and Control, Jinan, Shandong, China; 3 Department of Pharmacy, Shandong Provincial Hospital Affiliated to Shandong University, Jinan, Shandong, China; Universidad de Navarra, SPAIN

## Abstract

**Background and Aims:**

Liver stiffness (LS) and spleen stiffness (SS) are two most widely accessible non-invasive parameters for predicting esophageal varices (EV), but the reported accuracy of the two predictors have been inconsistent across studies. This meta-analysis aims to evaluate the diagnostic performance of LS and SS measurement for detecting EV in patients with chronic liver disease (CLD), and compare their accuracy.

**Methods:**

Pubmed/Medline, Embase, Cochrane Library and Ovid were searched for all studies assessing SS and LS simultaneously in EV diagnosis. A total of 16 studies including 1892 patients were included in this meta-analysis, and the pooled statistical parameters were calculated using the bivariate mixed effects models.

**Results:**

In detection of any EV, for LS measurement, the summary sensitivity was 0.83 (95% confidence interval [CI]: 0.78–0.87), and the specificity was 0.66 (95% CI: 0.60–0.72). While for SS measurement, the pooled sensitivity and specificity was 0.88 (95% CI: 0.83–0.92) and 0.78 (95% CI: 0.73–0.83). The summary receiver operating characteristic (SROC) curve values of LS and SS were 0.81 (95% CI: 0.77–0.84) and 0.88 (95% CI: 0.85–0.91) respectively, and the results had statistical significance (P<0.01). The diagnostic odds ratio (DOR) of SS (25.73) was significantly higher than that of LS (9.54), with the relative DOR value was 2.48 (95%CI: 1.10–5.60), P<0.05.

**Conclusions:**

Under current techniques, SS is significantly superior to LS for identifying the presence of EV in patients with CLD. SS measurement may help to select patients for endoscopic screening.

## Introduction

Esophageal varices (EV) are mainly induced by portal hypertension [1], which is one of the most common consequences of chronic liver disease (CLD). Variceal bleeding from rupture of EV is associated with high mortality [2]. According to the most recent guidelines [3], all patients with newly diagnosed cirrhosis are recommended to undergo screening esophagogastroduodenoscopy (EGD) for identifying varices. However, the invasive nature of EGD leads to significant healthcare costs and patient discomfort [4]. There is thus considerable interest in developing non-invasive methods with acceptable diagnostic accuracy to predict the presence and size of EV.

Several serum and radiological parameters have been put forward for predicting EV, such as serum fibrosis markers, liver stiffness (LS), spleen stiffness (SS), LS-spleen diameter to platelet ratio score [5–7]. Among them, it has been shown that both liver and spleen stiffness were more accurate in identifying EV and the degree of portal hypertension than other non-invasive parameters [8]. LS has been largely accepted to reflect the degree of fibrosis and the presence of EV in CLD. Several studies have revealed that LS measured by elastography may represent a useful non-invasive tool for predicting EV [9,10], notably in combination with other non-invasive parameters [11]. Current European Guidelines recommend to avoid screening EGD in patients with LS< 20kPa and platelet count >150,000 [12]. While the role of LS alone in predicting varices is controversial due to unsatisfactory diagnostic accuracy and lack of consistent results [3]. In the last few years, research emphasis has been placed on SS measurement in predicting EV and clinical significant portal hypertension. Portal hypertension leads to spleen congestion and fibrosis, which is sufficient to increase organ stiffness [13].

Recently, more and more studies have attempted to clarify the utility of SS and LS for EV diagnosis in patients with CLD, but the results have been controversial. Research has shown that SS assessed by elastography was a more effective parameter with high diagnostic accuracy for identifying and grading EV than LS [14,15]. Conversely, other studies have concluded that spleen elastography is not superior to liver elastography in predicting EV for its inconstant accuracy, poor repeatability and highly unreliable measurement [16–18]. In 2014, a meta-analysis summarized the accuracy of SS measurement in predicting EV. It showed that the SS measurement was acceptable, but had limited accuracy for EV diagnosis [19]. However, the diagnostic performance of SS compared with the conventional LS measurement is still uncertain.

In light of the uncertain utility of SS and LS in EV diagnosis, we conducted a systemic review and meta-analysis based on the increasing number of comparative studies. We evaluated the diagnostic performance of SS and LS simultaneously on same individuals in this meta-analysis, and compared the accuracy of the two parameters for predicting and grading EV in CLD.

## Materials and Methods

### Selection criteria

Studies were included if they met the following inclusion criteria: (1) performed in adults with CLD who did not undergo liver transplantation or transjugular intrahepatic portosystemic shunt (TIPS); (2) reported the performance of SS and LS measurement simultaneously, using the same elastography technique based on ultrasound or magnetic resonance; (3) used EGD as the reference standard for detecting and grading EV; (4) provided necessary data to calculate the true positive, false positive, true negative, and false negative value for both SS and LS on diagnosis of EV; (5) selected an optimum cut-off value to maximize sensitivity and specificity according to the receiver operating characteristic (ROC) or Youden Index. If such data presented in original articles were insufficient, the corresponding author would be contacted by e-mail to provide them. Studies without available relevant data after contacting original authors were excluded.

### Search strategy

A systematic search was performed through Pubmed/Medline, Embase, Cochrane Library and Ovid to identify all relevant studies assessing SS and LS simultaneously in EV diagnosis. Relevant studies published prior to 1 May 2016 were searched using the following keywords: spleen stiffness, liver stiffness, elastography, varices. A manual search was also carried out on reference lists of identified articles. All studies were limited to articles with an English abstract.

### Study selection and data extraction

Two investigators (X.M. and L.W.) independently screened the search results and reviewed relevant full texts to determine eligibility. Discrepancies were resolved in consultation with a senior reviewer (Q.Z.). For each included study, the following data were extracted: author, country, year of publication, study design, number of patients, age, gender, body mass index (BMI), etiology of CLD, proportion of cirrhosis, Child-Pugh score, prevalence of EV or severe EV, definition of severe EV, measuring techniques, invalid measurement, optimum cut-off value according to ROC curve or Youden Index, sensitivity, specificity and area under ROC curve for SS and LS respectively. We imputed the number of true positive, false positive, false negative and true negative results of SS and LS respectively on EV or severe EV diagnosis in all patients with EGD.

### Quality assessment

Risk of bias was assessed separately by two investigators using the revised Quality Assessment of Diagnostic Accuracy Studies (QUADAS-2) tool [20]. This tool is divided into 4 domains including patient selection, index test, reference standard, flow and timing. Each domain is assessed for risk of bias, and the first 3 domains are assessed for applicability as well. In this meta-analysis, LS and SS measurement were regarded as the index test, and the reference standard referred to EGD.

### Data synthesis and analysis

Based on extracted data, the summary sensitivities, specificities, and diagnosis odds ratio (DOR) with corresponding 95% confidence interval (CI) were calculated to evaluate the performance of liver and spleen stiffness measurements for EV and severe EV diagnosis. The DOR comprises a combination of sensitivity and specificity, and it was regarded as a single indicator of diagnostic test accuracy [21]. The summary ROC (SROC) curve was also performed as an alternative global measure of accuracy to avoid the influence of heterogeneity and different cut-off value. All summary parameters were calculated using the bivariate mixed effects models. In addition, using Fagan nomogram, we evaluated the post-test probabilities of EV on assumption of 57% pre-test probability following a positive or negative test result. To provide a clinically meaningful comparison, we conducted the SROC curve for both liver and spleen stiffness measurements simultaneously, and compared their area under SROC curve using Z-test [22]. We also calculated the relative DOR (rDOR) ratios with 95% CI of the two parameters. When 95% CI do not include the unity, the difference of DOR between tests is statistically significant.

Between-study heterogeneity was assessed by computing Higgin’s I^2^ and chi-square test (P value). An I^2^ value more than 50% or a P value less than 0.10 was considered substantial heterogeneity. Besides, we used meta-regression analyses according to different study characteristics to investigate sources of heterogeneity. Because there are considerable variations across different techniques for stiffness measurement and different stages of CLD, we also performed subgroup analyses to investigate the influence of such variability on diagnostic performance.

Deek’s funnel plot was used to test the presence of publication bias, in which a regression of diagnostic log odds ratio against 1/sqrt (effective sample size) and weighting by effective sample size was conducted, with a P value less than 0.10 suggesting significant asymmetry [23]. All statistical analyses were performed by STATA 12.0 (College Station, TX) software using MIDAS command. This meta-analysis was based on PRISMA (Preferred Reporting Items for Systematic Reviews and Meta-Analyses) checklist (S1 PRISMA Checklist).

## Results

### Search results

**Fig 1:**
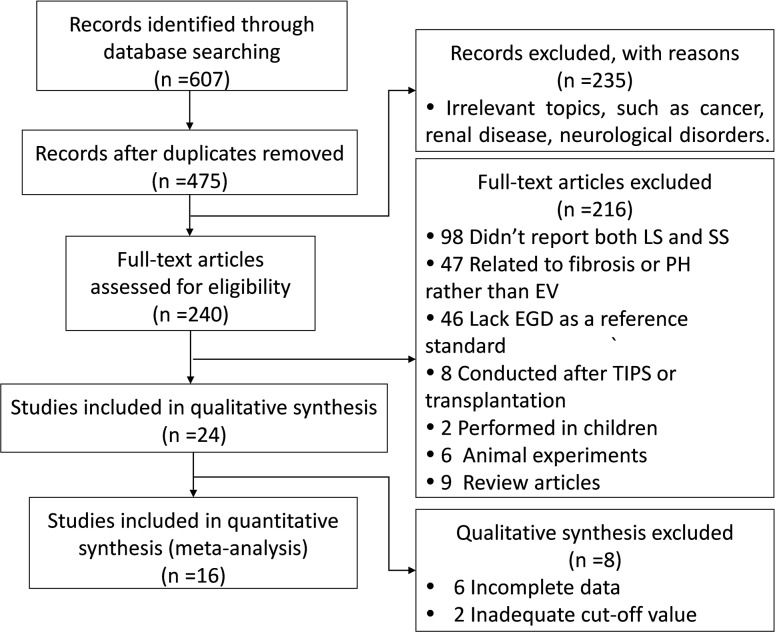
Flow diagram showing study identification and selection. LS, liver stiffness; SS, spleen stiffness; PH, portal hypertension; EV, esophageal varices; EGD, esophagogastroduodenoscopy; TIPS, transjugular intrahepatic portosystemic shunt.

A total of 607 studies were identified based on described search strategies. After removing duplicates and irrelevant articles, 240 studies were screened for further review. 98 studies were excluded because they didn’t report on both liver and spleen stiffness measurement, and 93 studies could not be included for not relevant to EV (n = 47) or lack of EGD (n = 46). 33 studies were excluded for children or animal subjects (n = 8), surgery experience (n = 8), incomplete data (n = 6), inadequate cut-off value (n = 2) or type of reviews (n = 9). Ultimately, a total of 16 studies (14 full-text studies and 2 abstracts) including 1892 patients in whom both SS and LS were measured for EV detection were selected for meta-analysis. 13 of these reported the diagnostic performance of SS and LS in identifying the presence of EV [8,14,18,24–33], while 5 studies were available in severe EV diagnosis through spleen and liver stiffness measurement [27,31,34–36]. The coefficient of agreement between the two investigators was very good. Fig 1 shows the flow diagram of study selection.

### Characteristics of included studies

The main characteristics of studies included in our meta-analysis are summarized in Table 1 and Table 2. In total, 1892 patients (median age, 56.4 years, 68.6% male) were included, of which the overall prevalence of EV and severe EV were 57.4% (10.0%-92.1%), 33.3% (27.1%-60.0%). 6 studies were performed on patients caused by viral hepatitis alone [8,14,18,24–25,27], and 13 studies referred to cirrhosis only [8,18,24,27–36]. As the most commonly used technique, transient elastography (TE) was used in 10 studies for liver and spleen stiffness measurement [8,14,18,24,25,27,29,30,32,36]. Another 4 techniques, acoustic radiation force impulse (ARFI) [26,34], virtual touch tissue quantification (VTTQ) [33], share wave elastography (SWE) and magnetic resonance elastography (MRE) [28,35,31] were used in other included studies. According to the QUADAS-2 scale, overall, studies were felt to be at low risk of bias and had good applicability (S1 Table).

**Table 1:** Baseline Characteristics of Studies and Patients Included in the Meta-analysis.

Study, Reference, Year	Country	Technique	Total patients	Mean age	Mean BMI	Gender %male	Cirrhosis (%)	Child score, A/B/C%	Etiology (%viral)
Al-Dahshan et al [24], 2012	Egypt	TE	60	52.6	NR	78.3	100	NR	100
Alsebaey et al (Ab) [25], 2015	Egypt	TE	165	NR	NR	NR	NR	NR	100
Attia et al [26], 2015	Germany	ARFI	78	54	NR	79.5	86	27/59/14	15
Bota et al [34], 2012	Romania	ARFI	145	59.1	26.7	60	100	46/43/11	50.3
Calvaruso et al [27], 2013	Italy	TE	112	63.2	27	69.8	100	100/0/0	100
Calvaruso et al (Ab) [18], 2010	Italy	TE	159	63	NR	71.7	100	NR	100
Colecchia et al [8], 2012	Italy	TE	113	54	25	71	100	68/32/0	100
Elkrief et al [35], 2015	France	SWE	79	55	26	78.5	100	30/25/44	45
Fraquelli et al [14], 2014	Italy	TE	132	52	23	59.1	23	NR	100
Grgurevic et al [28], 2015	Croatia	SWE	87	62.6	NR	78.2	100	51/29/20	25.3
Liu et al [29], 2013	China	TE	101	50.9	NR	64.9	100	76/19/5	57.5
Sharma et al [30], 2013	India	TE	200	49.3	24.6	88.5	100	32/57/11	29.9
Shin et al [31], 2014	South Korea	MRE	139	57.3	NR	73.4	100	NR	80.6
Stefanescu et al [32], 2011	Romania	TE	137	56	26.4	56.2	100	65/28/7	NR
Stefanescu et al [36], 2014	Romania	TE	90	55.7	26.7	55.6	100	62/36/1	20
Takuma et al [33], 2011	Japan	VTTQ	95	68.7	NR	48.4	100	NR	76.8

ARFI, acoustic radiation force impulse; BMI, body mass index; MRE, magnetic resonance elastography; NR, not reported; SWE, shear wave ultrasound elastography; TE, transient elastography; VTTQ, virtual touch tissue quantification.

**Table 2:** Characteristics of the Diagnostic Performance of LS and SS for Predicting EV in 16 Included Studies.

Study, Reference	Total patients (invalid measures)	No. of EV/SEV	Liver Stiffness							Spleen Stiffness						
			CUT-OFF	SEN	SPE	PPV	NPV	LR+	LR-	CUT-OFF	SEN	SPE	PPV	NPV	LR+	LR-
Al-Dahshan et al [24]	60 (NR)	EV:30	17.75 kPa	0.93	0.47	0.64	0.88	1.75	0.14	50.4 kPa	0.80	0.73	0.75	0.79	3.00	0.27
Alsebaey et al (Ab) [25]	165 (NR)	EV:55	20.4 kPa	0.82	0.72	0.59	0.89	2.90	0.25	43.2 kPa	0.93	0.84	0.74	0.96	5.67	0.09
Attia et al [26]	78 (0)	EV:59	2.45 m/s	0.93	0.79	0.93	0.79	4.43	0.09	2.63 m/s	0.98	0.89	0.97	0.94	9.34	0.02
Bota et al [34]	145 (L2/S3) ^a^	SEV:62	2.25 m/s	0.94	0.29	0.50	0.86	1.32	0.22	2.55 m/s	0.97	0.20	0.48	0.89	1.22	0.16
Calvaruso et al [27]	112 (16)	EV:54	17.0 kPa	0.70	0.57	0.68	0.6	1.64	0.52	50.0 kPa	0.65	0.60	0.67	0.57	1.60	0.59
	112 (16)	SEV:26	19.0 kPa	0.73	0.54	0.37	0.84	1.60	0.50	54.0 kPa	0.81	0.70	0.5	0.91	2.69	0.27
Calvaruso et al (Ab) [18]	159 (15)	EV:80	21 kPa	0.70	0.72	0.76	0.66	2.49	0.42	47 kPa	0.79	0.70	0.77	0.73	2.65	0.30
Colecchia et al [8]	113 (13)	EV:53	21.4 kPa	0.83	0.81	0.83	0.81	4.34	0.21	46 kPa	0.94	0.77	0.82	0.92	4.03	0.07
Elkrief et al [35]	79 (5)	SEV:45/46^b^	24.7 kPa	0.82	0.45	0.70	0.62	1.49	0.40	32.3 kPa	0.48	0.71	0.73	0.45	1.67	0.73
Fraquelli et al [14]	132 (22)	EV:11	19 kPa	0.73	0.47	0.13	0.94	1.38	0.57	65 kPa	0.91	0.80	0.33	0.99	4.50	0.11
Grgurevic et al [28]	87 (0)	EV:54	19.7 kPa	0.83	0.67	0.80	0.71	2.50	0.25	30.3 kPa	0.80	0.76	0.84	0.69	3.28	0.27
Liu et al [29]	101 (0)	EV:93	18.0 kPa	0.91	0.63	0.97	0.38	2.44	0.14	44.5 kPa	0.88	0.63	0.96	0.31	2.35	0.19
Sharma et al [30]	200 (26)	EV:124	27.3 kPa	0.86	0.70	0.89	0.77	3.25	0.12	40.8 kPa	0.85	0.79	0.91	0.84	3.93	0.07
Shin et al [31]	139 (0)	EV:78	4.58 kPa	0.91	0.72	0.79	0.80	2.91	0.20	7.23 kPa	0.94	0.76	0.84	0.8	3.97	0.20
	139 (0)	SEV:45	4.81 kPa	0.60	0.72	0.49	0.91	2.04	0.20	7.60 kPa	0.76	0.66	0.52	0.85	2.22	0.37
Stefanescu et al [32]	137 (NR)	EV:116	28 kPa	0.74	0.62	0.91	0.30	1.95	0.42	46.4 kPa	0.84	0.71	0.94	0.44	2.93	0.23
Stefanescu et al [36]	90 (0)	SEV:47	38 kPa	0.89	0.56	0.70	0.62	2.13	0.56	53 kPa	0.89	0.51	0.67	0.81	1.83	0.21
Takuma et al [33]	95 (0)	EV:40	2.33 m/s	0.75	0.58	0.61	0.72	1.78	0.43	3.43 m/s	0.88	0.96	0.95	0.90	19.69	0.13

EV, esophageal varices; SEV, severe esophageal varices; SEN, sensitivity; SPE, specificity; PPV, positive predictive value; NPV, negative predictive value; LR+, positive likelihood value; LR-, negative likelihood value; NR, not reported.

^a^ Valid ARFI measurements in the liver in 143/145 patients, and in the spleen in 142/145 patients.

^b^ 45 SEV in patients with valid LS measurement, while 46 SEV in patient with valid SS measurement.

### Diagnostic accuracy of liver stiffness for the prediction of esophageal varices

**Fig 2:**
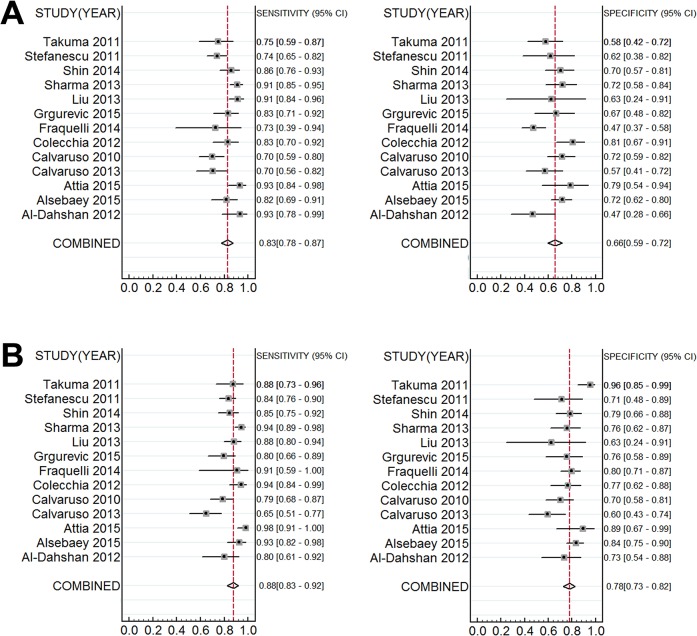
Forest plot of individual study evaluates of sensitivity and specificity for any esophageal varices diagnosis. The base vertical imaginary line indicates the combined effects. (A) Accuracy of liver stiffness measurement for estimating the presence of esophageal varices. (B) Accuracy of spleen stiffness for detecting the presence of any esophageal varices in chronic liver disease.

**Fig 3:**
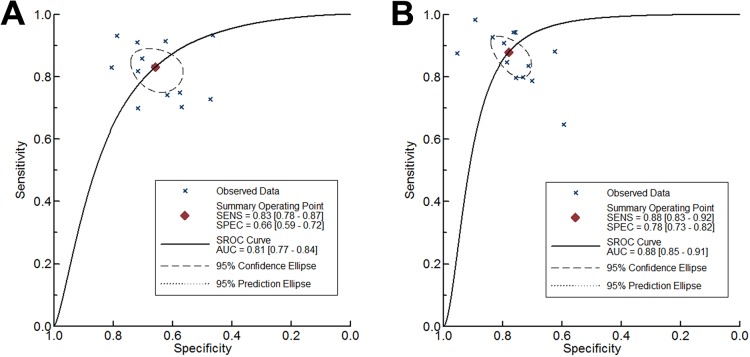
Summary receiver operating characteristic (SROC) curve of sensitivity versus specificity. (A) SROC curve of liver stiffness for prediction of any esophageal varices. (B) SROC curve of spleen stiffness for detecting the presence of esophageal varices.

**Fig 4:**
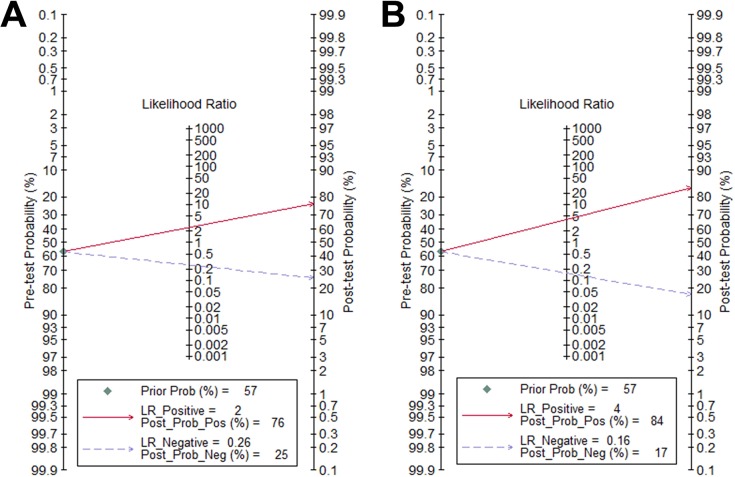
Fagan plot analysis to evaluating the clinical utility of liver and spleen stiffness for diagnosis of esophageal varices. (A) For LS measurements, with a pre-test probability of EV of 57%, the post-test probability of EV, given negative and positive results, were 25% and 76%. (B) For SS measurements, with a pre-test probability of EV of 57%, the post-test probability of EV, given negative and positive results, were 17% and 84%.

The diagnostic accuracy of liver and spleen stiffness measurement for prediction of the presence of EV was evaluated in 13 studies [8,14,18,24–27,28–33]. For LS measurement, the summary sensitivity was 0.83 (95% CI: 0.78–0.87), the summary specificity was 0.66 (95% CI: 0.60–0.72) (Fig 2A), the summary positive likelihood ratio (LR+) was 2.44 (95% CI: 1.99–2.99), the summary negative likelihood ratio (LR-) was 0.26 (95% CI: 0.19–0.35), the summary DOR was 9.54 (95% CI: 5.85–15.56), and the area under SROC curve was 0.81 (95% CI: 0.77–0.84) (Fig 3A). When the pre-test probability of EV was 57%, according to the Fagan plot analysis, LS was able to increase the post-probability to 76% following a positive result and lower the probability of to 25% with a negative measurement (Fig 4A).

Minor heterogeneity has been observed between studies on LS measurement, with I^2^ = 41.76%, P = 0.09. There was not significant threshold effect between studies, with the Spearman correlation coefficient = -0.17, P = 0.58. According to meta-regression analyses, basic characteristics of patients could explain the source of heterogeneity. Studies with the mean age less than 55 years old showed higher diagnostic accuracy compared to those performed in older patients. Research involving more male participants (over 70%) also improved the diagnostic performance of LS (P<0.01). The accuracy of LS was not affected by technique for measurement, location, quality of study, proportion of cirrhosis, etiology of disease, sample size (P>0.05) (S2 Table). Funnel plot asymmetry test demonstrated that there was no evidence of publication bias between studies (P = 0.68).

Separate analysis specific to TE technique (n = 9) was conducted to demonstrate the optimism range of cut-off value. In terms of DOR value, there were not significant differences between studies with the cut-off value lower than 21 kPa (n = 5, DOR = 7.21) and the others (n = 4, DOR = 11.523), P = 0.09.

### Diagnostic accuracy of spleen stiffness for the prediction of esophageal varices

For SS measurement, the summary sensitivity was 0.88 (95% CI: 0.83–0.92), the summary specificity was 0.78 (95% CI: 0.73–0.83) (Fig 2B), the summary LR+ was 4.00 (95% CI: 3.11–5.15), the summary LR- was 0.16 (95% CI: 0.10–0.23), the summary DOR was 25.73 (95% CI: 13.74–48.19), and the area under SROC curve was 0.88 (95% CI: 0.85–0.91) (Fig 3B). According to the Fagan plot analysis, when there was 57% pre-test probability of EV, a negative SS measurement could decrease the post-probability to as low as 17%, while a positive result indicated 84% probability of having EV (Fig 4B).

There was not significant heterogeneity in the analysis of SS for the prediction of EV (P = 0.13, I^2^ = 27.76%). Threshold effect was not observed for SS analysis (P = 0.02). Funnel plot asymmetry test demonstrated that there was no evidence of publication bias for SS in EV diagnosis (P = 0.92).

Separate analysis specific to TE technique (n = 9) was conducted to demonstrate the optimism range of cut-off value. For studies with the cut-off values lower than 47 kPa, the DOR value of SS in predicting EV was 34.92, which is significantly higher than other studies with the cut-off value≥47 kPa, P<0.05.

### Spleen stiffness is superior to liver stiffness for the prediction of esophageal varices in patients with chronic liver disease

Our results indicated that SS predicted the presence of EV better than LS, on both sensitivity and specificity. The area under SROC curve of SS for diagnosis of EV was 0.88 (95% CI: 0.85–0.91), while the LS had a value of 0.81 (95% CI: 0.77–0.84) (Fig 3). There was significant difference between the two SROC values according to Z-test (Z = 3.74, P<0.01). The summary DOR of SS (DOR = 25.73) was higher than that of LS (DOR = 9.54), and the difference was statistical significant (rDOR = 2.48, 95% CI: 1.10–5.60, P = 0.03). Because the technique for measurement varies between included studies, a certain cut-off value could not be concluded accurately. To decrease the influence of different diagnostic thresholds, all included studies defined the optimum cut-off value according to the ROC curve or Youden index to maximize the sensitivity and specificity. At corresponding cut-off value, the summary sensitivity of SS and LS for detecting the presence of EV were 0.88 and 0.83 respectively (Z = 1.13, P = 0.26), whereas the specificity of SS was significantly higher than that of LS with the value of 0.78 and 0.66 (Z = 2.35, P = 0.02). A Z-test based on the joint model of sensitivity and specificity demonstrated that the diagnostic accuracy of SS and LS differed significantly for prediction of EV (P = 0.03). Table 3 summarized the pooled accuracy and the comparison of LS and SS measurement.

**Table 3:** Comparison of LS and SS for the prediction of EV and severe EV.

Statistical parameters	Prediction of any EV (13 studies)			Prediction of severe EV (5 studies)		
	LS	SS	Comparison	LS	SS	Comparison
Sensitivity (95%CI)	0.83 (0.78–0.87)	0.88 (0.83–0.92)	P = 0.26	0.82 (0.69–0.91)	0.83 (0.61–0.94)	P = 0.99
Specificity (95%CI)	0.66 (0.60–0.72)	0.78 (0.73–0.83)	P = 0.02 *	0.52 (0.39–0.65)	0.57 (0.37–0.75)	P = 0.75
Area under SROC curve (95% CI)	0.81 (0.77–0.84)	0.88 (0.85–0.91)	P<0.01 **	0.72 (0.68–0.76)	0.75 (0.71–0.79)	P = 0.32
Diagnostic odds ratio (95% CI)	9.54 (5.85–15.56)	25.73 (13.74–48.19)	rDOR = 2.48 (95%CI:1.10–5.60)P = 0.03*	4.98 (3.13–7.94)	6.47 (3.63–11.54)	rDOR = 1.31 (95%CI:0.60–2.89)P = 0.64

Z-test was used to compare the SROC and DOR value between LS and SS. CI, confidence interval; LS, liver stiffness; SS, spleen stiffness; SROC, summary receiver operating characteristic; rDOR, relative diagnostic odds ratio.

*P<0.05

**P<0.01

### Sensitivity Analysis

For EV identification, on restricting analysis to 9 studies performed with TE alone, the pooled sensitivity and specificity of LS were 0.83 (95%CI: 0.75–0.88) and 0.65 (95% CI: 0.56–0.72), while SS has the sensitivity and specificity of 0.87 (95% CI: 0.81–0.92) and 0.75 (95% CI: 0.69–0.80). The DOR value of SS (20.59) is still higher than LS measurement (8.61). For both LS and SS, sensitivity analysis after excluding non-cirrhosis (not included cirrhosis patients only), non-viral etiology (not caused by viral hepatitis only) and low quality studies (with high risk of bias according to QUADAS-2), did not significantly alter the primary results.

### Diagnostic accuracy of liver and spleen stiffness for the prediction of severe esophageal varices

5 studies including 567 cirrhosis patients provided sufficient data to assess the diagnostic performance of spleen and liver stiffness for identifying severe EV. Severe EV were defined as grade 2 or grade 3 varices in 3 included studies, while they were regarded as grade 2 or 3, or varices with the red color sign in the other study.

For LS measurement, the summary sensitivity was 0.82 (95% CI: 0.69–0.91), the summary specificity was 0.52 (95% CI: 0.39–0.65) (S1A Fig), the summary DOR was 4.98 (95% CI: 3.13–7.94), and the area under SROC curve was 0.72 (95% CI: 0.68–0.76). For SS measurement, the summary sensitivity and specificity for detecting severe EV was 0.83 (95% CI: 0.61–0.94) and 0.57 (95% CI: 0.37–0.75) respectively (S1B Fig), the summary DOR was 6.47 (95% CI: 3.63–11.54), and the area under SROC curve was 0.75 (95% CI: 0.71–0.79). The DOR of SS and LS did not differ significantly for detecting severe EV (rDOR = 1.31, 95% CI: 0.60–2.89).

Significant heterogeneity was observed in the analysis of severe EV. Because of the limited number of included studies, meta-regression could not be used to explore the factors inducing heterogeneity. Funnel plot asymmetry test demonstrated that there was no publication bias for LS and SS in detecting severe EV, with P = 0.15 and 0.55.

## Discussion

LS and SS are two non-invasive parameters receiving the most attention for identifying patients suffered from EV, but the diagnostic value of these two predictors is still controversial. In this meta-analysis, we evaluated the performance of LS and SS simultaneously for detecting EV and severe EV in patients with CLD, and compared their diagnostic accuracy. Our results indicated that SS was superior to LS for predicting the presence of EV in patients with CLD, while the diagnostic accuracy of both LS and SS were limited in predicting severe EV.

During the progression of liver cirrhosis and portal hypertension, passive congestion and tissue hyperplasia characterized by a combination of angiogenesis and fibrogenesis frequently occur in the spleen [37]. All these changes result in increased SS, which is closely related to portal hypertension and reflects the extra-hepatic hemodynamic changes. When it comes to LS, although it appears to be a reliable surrogate for liver biopsy in identifying mild or advanced fibrosis, the pathophysiological basis for its correlation with portal hypertension remains poorly defined [38]. It is clear that LS only reflects the increased intra-hepatic vascular resistance, but not the hyperdynamic circulation and the opening of portal-systemic shunts [38]. For this reason, SS predicts the formation of EV caused by splanchnic hemodynamics changes better than LS [8], which is consistent with our results.

Combination of different non-invasive markers is also an important and valid approach to exclude EV in clinical practice. It is considered that the combination of LS value with other spleen-related parameters results in an increased diagnostic accuracy [8]. This phenomenon indicates that the association of LS and parameters reflecting the extra-hepatic hemodynamic could be a valuable tool with better diagnostic accuracy for the prediction of EV. Studies have shown that combining the LS and SS measurements further increased the diagnostic accuracy of EV [30,32]. Hence, it is possible to construct a combinative model with satisfactory accuracy for predicting EV based on the SS measurement.

Several techniques were enrolled in our studies for liver and spleen stiffness measurement. As the most widely used method for organ stiffness assessment, TE is available in many clinical centers, although it requires a dedicated Fibroscan device [39]. It should be mentioned that the reliable measurements by TE is quite low in obese cases and patients with ascites. We observed that these kinds of cases were tend to be avoided in most original studies involved in this meta-analysis. In contrast, ARFI and SWE are two novel, popular, ultrasound technique based technologies, which could be used in the existence of ascites. However, there is limited validation of these two techniques and the measures of quality are not well defined [19]. In this meta-analysis, we observed that there was no significant heterogeneity between different techniques, and the threshold effect was not obvious. Thus, the diagnostic performance of all these techniques were comparable. Moreover, we excluded studies with a different threshold standard. All studies included in our meta-analysis determined its own cut-off value following the accordant standard, which minimizes the influence of different techniques and cut-off values and ensures the comparability of the studies.

For severe EV, our results indicated that both liver and spleen stiffness measurement showed limited diagnostic accuracy. From the current studies, LS is considered not to correlate with the grades of EV [40,41], whereas the SS measurement may be possible to identify severe EV, but the accuracy is not high [42]. Certainly, additional studies are needed to verify the diagnostic performance of LS and SS in predicting severe EV.

Singh et al summarized the accuracy of SS measurement as a new predictor in detection of EV [19]. Extending upon previous studies, we compared the diagnostic value of this new proposed parameter with the conventional LS measurement in the prediction of EV. We concluded that SS is significantly superior to LS in EV diagnosis, which is helpful in clinical practice. Besides, with the development of elastography techniques, more recent studies (especially in last two years) were involved in this meta-analysis, which keeps our study novel and timely. Thus, only 5 studies included in our meta-analysis were involved in the previous publication.

The strengths of our study were the comprehensive and simultaneous assessment of the diagnostic value of LS and SS for the prediction of EV, and provided an authentic comparison of the two useful parameters. All comparative studies included in our meta-analysis provided sufficient data for both LS and SS simultaneously, which was able to decrease the risk of bias from patient spectrum, disease prevalence and inter-observer variability. Furthermore, a Z-test was used to compare the SROC value of LS and SS for predicting the presence of EV, and the rDOR was also conducted to compare the diagnostic accuracy based on the DOR value, which confirms the reliability of our study.

The limitations of our meta-analysis should be taken into consideration. First, only 5 studies described the performance of SS and LS for severe EV diagnosis, which limited the conduction of meta-regression and subgroup analysis for explaining the heterogeneity. More research is also needed to validate our summary results of LS and SS in identifying severe EV. Second, minor heterogeneity existed in the analysis of LS for prediction of EV in our meta-analysis. Although the heterogeneity is acceptable and could be explained by characteristics of involved patients, it also affected the reliability of our results. Third, the range of detection and units are completely different regarding variety of included techniques, which limited their comparisons. Because all studies involved in this analysis have to report the performance of LS and SS simultaneously, the included number of some clinical frequently-used techniques, such as ARFI, SWE, was too small to be analyzed separately. For this reason, we could not obtain the optimism cut-off range of each technique. In this meta-analysis, only separate analysis specific to TE was provided. Therefore, our summary conclusion that SS is superior to LS for predicting the presence of EV also needs to be validated under specific techniques respectively based on more original studies in future.

In conclusion, our meta-analysis demonstrated that SS is superior to LS for predicting the presence of EV in patients with CLD. Although the accuracy of the two parameters in identifying severe EV is not high, they still could be considered as a choice for screening EV in newly diagnosed cirrhosis. Combination of LS and LS may improve the diagnostic accuracy, and it is also possible to construct a novel combinative model with higher accuracy in predicting EV. Simple, low-cost and more accurate non-invasive models are needed in future as surrogates of endoscopy for EV detection.

## Supporting Information

S1 PRISMA Checklist PRISMA (Preferred Reporting Items for Systematic Reviews and Meta-Analyses) checklist for this meta-analysis. (DOC)

S1 Dataset All relevant data within this paper are available in dataset. (XLS)

S1 Fig Forest plot of individual study evaluates of sensitivity and specificity for severe EV diagnosis. (A) Accuracy of liver stiffness measurement for estimating severe esophageal varices. (B) Accuracy of spleen stiffness for detecting severe esophageal varices in chronic liver disease.
(TIF)

S1 Table Quality assessment of studies included in the analysis (QUADAS 2). (DOC)

S2 Table Subgroup analysis reporting the diagnostic performance of LS for the detection of esophageal varices. (DOC)

## Abbreviations

ARFI: acoustic radiation force impulse
CI: confidence interval
CLD: chronic liver disease
DOR: diagnostic odds ratio
EGD: esophagogastroduodenoscopy
EV: esophageal varices
LR+: positive likelihood ratio
LR-: negative likelihood ratio
LS: liver stiffness
MRE: magnetic resonance elastography
NPV: negative predictive ratio
PPV: positive predictive ratio
rDOR: relative diagnostic odds ratio
ROC: receiver operator curve
SROC: summary receiver operator curve
SS: spleen stiffness
SWE: share wave elastography
TE: transient elastography
VTTQ: virtual touch tissue quantification.
