# Supplementary figures and images for: Spleen Stiffness Is Superior to Liver Stiffness for Predicting Esophageal Varices in Chronic Liver Disease: A Meta-Analysis
Source: PLoS One. 2016 Nov 9;11(11):e0165786. doi: 10.1371/journal.pone.0165786 (PMC5102398; doi:10.1371/journal.pone.0165786)

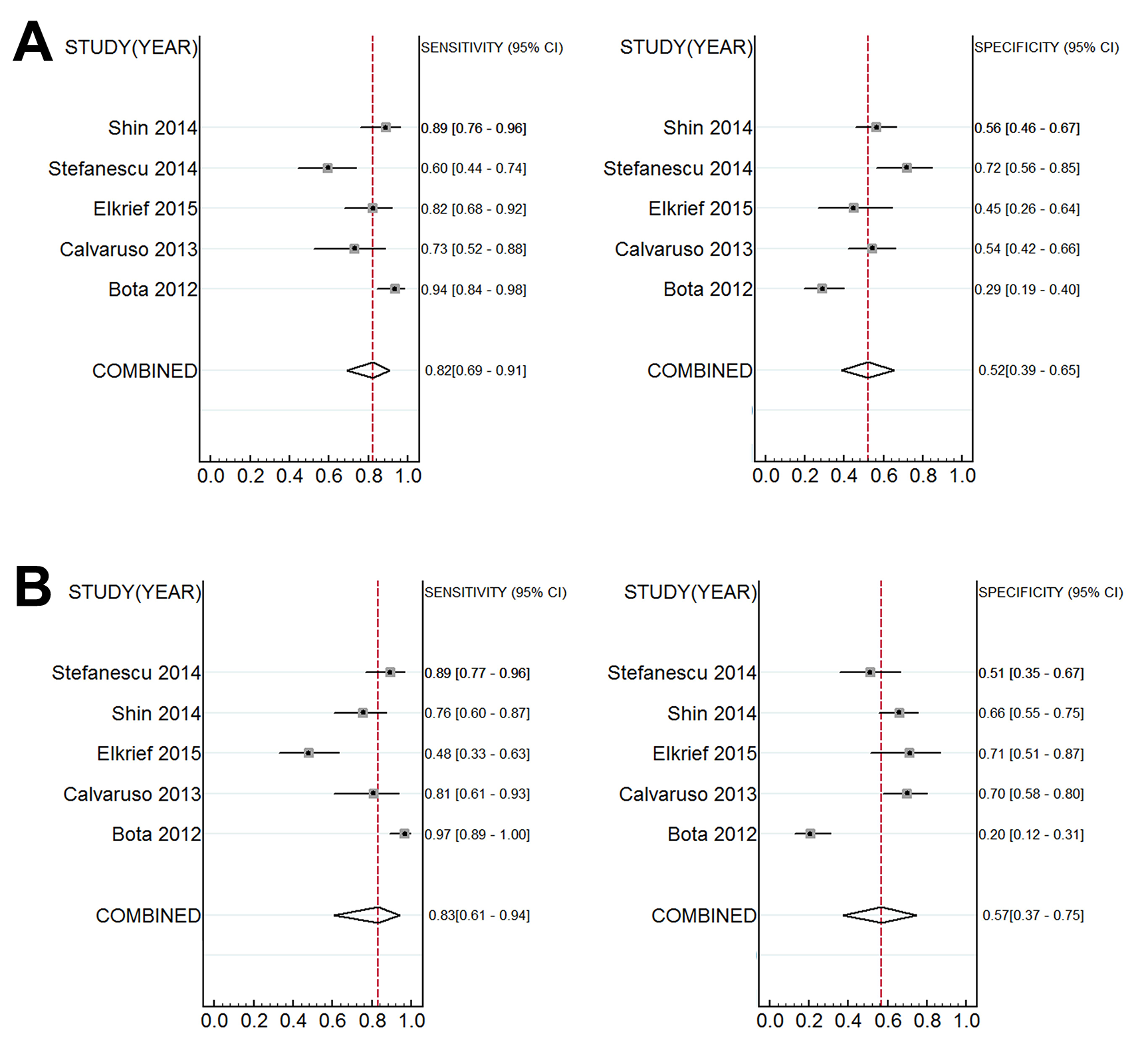

Supplement: S1 Fig — (A) Accuracy of liver stiffness measurement for estimating severe esophageal varices. (B) Accuracy of spleen stiffness for detecting severe esophageal varices in chronic liver disease. (TIF) [file pone.0165786.s003.tif]
