# Supplementary material for: Spleen Stiffness Is Superior to Liver Stiffness for Predicting Esophageal Varices in Chronic Liver Disease: A Meta-Analysis
Source: PLoS One. 2016 Nov 9;11(11):e0165786. doi: 10.1371/journal.pone.0165786 (PMC5102398; doi:10.1371/journal.pone.0165786)
Supplement: S1 Table — (DOC) [file pone.0165786.s004.doc]

**Supplementary Material**

| **Study, Reference, Year** |  | **Risk of bias** | | | |  |  | **Applicability concerns** | | |  |
| --- | --- | --- | --- | --- | --- | --- | --- | --- | --- | --- | --- |
|  | **Patients selection** | | **Index test** | **Reference standard** | **Folw and timing** | | **Patients selection** | | **Index test** | **Reference standard** | |
| Al-Dahshan et al,^24^ 2012 | High | | Unclear | Low | Low | | High | | Low | Low | |
| Alsebaey et al (Ab),^25^ 2015 | Low | | Low | Low | Low | | Low | | Low | Low | |
| Attia et al,^26^ 2015 | Low | | Low | Low | Low | | Low | | Low | Low | |
| Bota et al,^34^ 2012 | Low | | Low | Low | Low | | Low | | Low | Low | |
| Calvaruso et al,^27^ 2013 | Low | | Unclear | Low | Low | | Low | | Low | Low | |
| Calvaruso et al (Ab),^18^ 2010 | Low | | Unclear | Low | Low | | Low | | Low | Low | |
| Colecchia et al,^8^ 2012 | Low | | Low | Low | Low | | Low | | Low | Low | |
| Elkrief et al,^35^ 2015 | Low | | Low | Low | Low | | Low | | Low | Low | |
| Fraquelli et al,^14^ 2014 | Low | | Low | Low | High | | Low | | Low | Low | |
| Grgurevic et al,^28^ 2015 | Low | | Unclear | Low | Low | | Low | | Low | Low | |
| Liu et al,^29^ 2013 | Low | | Unclear | Low | Low | | Low | | Low | Low | |
| Sharma et al,^30^ 2013 | Low | | Low | Low | Low | | Low | | Low | Low | |
| Shin et al,^31^ 2014 | Low | | Low | Low | Low | | Low | | Low | Low | |
| Stefanescu et al,^32^ 2011 | Low | | Unclear | Low | Low | | Low | | Low | Low | |
| Stefanescu et al,^36^ 2014 | Low | | Low | Low | Low | | Low | | Low | Low | |
| Takuma et al,^33^ 2011 | Low | | Unclear | Low | Low | | Low | | Low | Low | |

**S1 Table. Quality assessment of studies included in the analysis (QUADAS 2)**
