# Supplementary material for: Spleen Stiffness Is Superior to Liver Stiffness for Predicting Esophageal Varices in Chronic Liver Disease: A Meta-Analysis
Source: PLoS One. 2016 Nov 9;11(11):e0165786. doi: 10.1371/journal.pone.0165786 (PMC5102398; doi:10.1371/journal.pone.0165786)
Supplement: S2 Table — (DOC) [file pone.0165786.s005.doc]

|  |  |  |  |  |  |  |  |  |  |
| --- | --- | --- | --- | --- | --- | --- | --- | --- | --- |
|  |  |  |  |  |  |  |  |  |  |
|  |  |  |  |  |  |  |  |  |  |
|  |  |  |  |  |  |  |  |  |  |
|  |  |  |  |  |  |  |  |  |  |
|  |  |  |  |  |  |  |  |  |  |
|  |  |  |  |  |  |  |  |  |  |
|  |  |  |  |  |  |  |  |  |  |
|  |  |  |  |  |  |  |  |  |  |
|  |  |  |  |  |  |  |  |  |  |
|  |  |  |  |  |  |  |  |  |  |
|  |  |  |  |  |  |  |  |  |  |
|  |  |  |  |  |  |  |  |  |  |
|  |  |  |  |  |  |  |  |  |  |
|  |  |  |  |  |  |  |  |  |  |
|  |  |  |  |  |  |  |  |  |  |
|  |  |  |  |  |  |  |  |  |  |

**Supplementary Material**

**S2 Table. Subgroup analysis reporting the diagnostic performance of LS for the detection of esophageal varices**

| **Variable** | **Subgroups** | **N** | **Sensitivity** | **I^2^** | **Specificity** | **I^2^** | **LR+** | **LR-** | **DOR** | **rDOR** | **P value** |
| --- | --- | --- | --- | --- | --- | --- | --- | --- | --- | --- | --- |
| **Mean age** | ≤ 55 | 6 | 0.90(0.86-0.93) | 35 | 0.65(0.52-0.77) | 81 | 2.58(1.79-3.72) | 0.15(0.11-0.22) | 17.06(9.05-32.14) | 2.54(0.76-8.43) | P=0.11 |
|  | > 55 | 6 | 0.77(0.71-0.81) | 42 | 0.65(0.59-0.71] | 0 | 2.21(1.82-2.68) | 0.36(0.28-0.46) | 6.14(4.04-9.34) |
| **Male %** | ≤ 70% | 5 | 0.79(0.67-0.87) | 69 | 0.54(0.45-0.62) | 9 | 1.69(1.41-2.03) | 0.40(0.26-0.62) | 4.25(2.37-7.60) | 3.35(1.68-6.67) | P<0.01** |
|  | > 70% | 7 | 0.87(0.80-0.92) | 74 | 0.70(0.63-0.76) | 49 | 2.88(2.36-3.52) | 0.19(0.13-0.29) | 15.17(9.34-24.65) |
| **Location** | Asia & Egypt | 6 | 0.87(0.82,0.91) | 55 | 0.66(0.58,0.73) | 47 | 2.55(2.04,3.19) | 0.19(0.13,0.27) | 13.34(8.07,22.06) | 1.35(0.40-4.56) | P=0.59 |
|  | Europe | 7 | 0.78(0.70,0.85) | 61 | 0.67(0.57,0.77) | 77 | 2.39(1.65,3.46) | 0.33(0.21,0.51) | 7.32(3.34,16.04) |
| **Technique** | TE | 9 | 0.83(0.75,0.88) | 75 | 0.65(0.56,0.72) | 73 | 2.33(1.83,2.98) | 0.27(0.19-0.40) | 8.61(4.89-15.19) | 1.31(0.41-4.19) | P=0.62 |
|  | Others | 4 | 0.85(0.77,0.91) | 54 | 0.68(0.58,0.77) | 14 | 2.68(1.92,3.74) | 0.22( 0.13,0.36) | 12.42(5.51,28.02) |
| **Etiology** | Viral | 6 | 0.79(0.71-0.85) | 50 | 0.64(0.53-0.74) | 81 | 2.17(1.61-2.92) | 0.33(0.23-0.48) | 6.52(3.57-11.92) | 1.50(0.50-4.54) | P=0.43 |
|  | Mixed | 7 | 0.86(0.80,0.91) | 76 | 0.68(0.61,0.75) | 0 | 2.69(2.08,3.46) | 0.21(0.13,0.32) | 13.12(6.80,25.30) |
| **Cirrhosis %** | 100% | 10 | 0.83(0.77-0.88) | 74 | 0.66(0.60-0.72) | 41 | 2.46(2.02-2.98) | 0.26(0.19-0.36) | 9.45(5.89-15.18) | 1.17(0.31-4.39) | P=0.79 |
|  | <100% | 3 | 0.86(0.79-0.92) | 61 | 0.62(0.55-0.68) | 87 | 2.46(1.25-4.83) | 0.23(0.09-0.58) | 11.02(2.55-47.62) |
| **Study quality** | Low | 2 | 0.88(0.74-0.96) | 65 | 0.48(0.38-0.56) | 0 | 1.59(1.22-2.07) | 0.31(0.08-1.24） | 5.157(1.05-25.38) | 4.95(0.8-30.49) | P=0.08 |
|  | High | 11 | 0.83(0.77,0.88) | 74 | 0.70(0.65-0.74) | 13 | 2.76(2.29-3.33) | 0.24(0.18-0.34) | 11.38(6.97-18.57) |
| **Sample size** | ≤100 | 6 | 0.84(0.76-0.89) | 66 | 0.65(0.55-0.75) | 64 | 2.41(1.75-3.33) | 0.25(0.16-0.41) | 9.61(4.54-20.34) | 0.97(0.33-2.87) | P=0.95 |
|  | >100 | 7 | 0.83(0.74,0.89) | 77 | 0.67(0.58-0.74) | 71 | 2.47(1.88-3.24) | 0.26(0.17-0.41) | 9.39(4.78-18.45) |

LR+, positive likelihood ratio; LR+, negative likelihood ratio; DOR, diagnostic odds ratio; rDOR, relative diagnostic odds ratio.
